# Polypharmacology: promises and new drugs in 2022

**DOI:** 10.1007/s43440-023-00501-4

**Published:** 2023-06-06

**Authors:** Piotr Ryszkiewicz, Barbara Malinowska, Eberhard Schlicker

**Affiliations:** 1 Department of Experimental Physiology and Pathophysiology, Medical University of Bialystok, 15-222 Bialystok, Poland; 2 Department of Pharmacology and Toxicology, University of Bonn, 53127 Bonn, Germany

**Keywords:** Polypharmacology, Multi-target drugs, Multi-target-directed ligands, Targeted therapy, Polytherapy

## Abstract

Polypharmacology is an emerging strategy of design, synthesis, and clinical implementation of pharmaceutical agents that act on multiple targets simultaneously. It should not be mixed up with polytherapy, which is based on the use of multiple selective drugs and is considered a cornerstone of current clinical practice. However, this ‘classic’ approach, when facing urgent medical challenges, such as multifactorial diseases, increasing resistance to pharmacotherapy, and multimorbidity, seems to be insufficient. The ‘novel’ polypharmacology concept leads to a more predictable pharmacokinetic profile of multi-target-directed ligands (MTDLs), giving a chance to avoid drug-drug interactions and improve patient compliance due to the simplification of dosing regimens. Plenty of recently marketed drugs interact with multiple biological targets or disease pathways. Many offer a significant additional benefit compared to the standard treatment regimens. In this paper, we will briefly outline the genesis of polypharmacology and its differences to polytherapy. We will also present leading concepts for obtaining MTDLs. Subsequently, we will describe some successfully marketed drugs, the mechanisms of action of which are based on the interaction with multiple targets. To get an idea, of whether MTDLs are indeed important in contemporary pharmacology, we also carefully analyzed drugs approved in 2022 in Germany: 10 out of them were found multi-targeting, including 7 antitumor agents, 1 antidepressant, 1 hypnotic, and 1 drug indicated for eye disease.

## Introduction

Polypharmacotherapy (combination therapy, polytherapy) is a contemporary treatment strategy for multifactorial conditions such as metabolic syndrome, type II diabetes, hypertension, psychiatric or neurodegenerative disorders, and cancer. The World Health Organization (WHO) defines polypharmacotherapy as the *safe, effective, and evidence-based treatment of patients with at least five drugs* [1–4]. In common parlance, a therapeutic regimen conducted with at least two drugs is also considered polytherapy. Supported by reliable scientific data and conducted in accordance with the principles of evidence-based medicine (EBM), polypharmacotherapy is a foundation of current clinical practice.

The continuous progress in medical and life sciences, especially within the omics sciences (genomics, proteomics, metabolomics, etc.) gave the perspective for a new strategy for drug design and treatment, which was termed polypharmacology [5–10]. According to the National Library of Medicine (2014), this promising novel concept is defined as *the design or use of pharmaceutical agents that act on multiple targets or disease pathways* [11]; this definition is still actual [e.g., 5–10]. However, polypharmacology does not compete with polytherapy and should not be considered as a phenomenon exclusive to well-established practices [8]. In fact, it opens new doors for meeting urgent medical needs by the rational use of the promiscuous nature of multi-target-directed ligands (MTDLs) in order to obtain desired synergistic effects on altered biochemical pathways [8, 9].

## From monospecific ligands to MTDLs

**Fig. 1:**
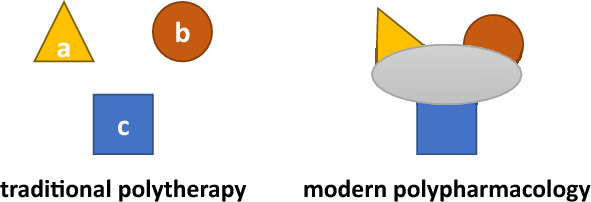
General comparison of traditional polypharmacotherapy and the modern concept of polypharmacology. The individual geometric figures (a, b, c) illustrate substances traditionally used as components of polytherapy for a specific condition (e.g., hypertension). Each of them exhibits only one activity in vivo. Combining activities of all these components (a, b, c) in a single multi-target-directed ligand (MTDL) corresponds to the concept of polypharmacology (based on: Proschak et al. 2019 [7])

The goal of the classical concept of designing new molecules with the potential to become therapeutic substances was to find a lead structure that would be able to bind to only one certain target–an enzyme, a receptor, etc. [12]. This approach can thus be summarized as one drug affecting one molecular target, the modulation of which triggers one measurable pharmacodynamic effect (optimally proportional to the dose used) [13]. The lack of affinity of the drug to targets besides the main one was considered its major advantage, as this was supposed to grant a low risk of side effects [6, 7]. However, the latest progress in biochemical and molecular sciences suggests that this approach is too simplistic for the synthesis of modern drugs for multifactorial diseases. Constant exploration for still unidentified components of the pathways of complex diseases prompted investigators to define correlations between the pathways at the molecular level and resulted in the rapid growth of the so-called “network pharmacology” concept [8, 9, 14]. Paradoxically, what was considered to be a feature significantly reducing the safety potential of new molecules with intended therapeutic use, i.e., the off-target effects (resulting from the affinity of a given molecule to targets other than those predicted), became the driving force behind the development of an entirely new concept. The random and serendipitous discovery of new targets after drugs had already been approved tends to turn into a fully controlled process, carried out a long time before the registration procedure begins [8, 15]. After all, the promiscuity of a ligand (i.e., the ability to bind to more than one molecular target) ceases to be a disadvantage once its activities are predicted early enough. This promiscuity may even become its greatest advantage, provided that these additional activities, carefully planned using the advances of molecular sciences and increasingly accurate molecular modeling methods, produce a beneficial synergistic or additive effect on the altered biochemical pathways. This is how the idea of polypharmacology was born. As stated above, it includes synthesis and eventual implementation into clinical practice of compounds with precisely planned multi-target activity. At its core (Fig. 1) is the assumption that in the case of the treatment of multifactorial diseases, such as multiple sclerosis [16], Alzheimer’s disease [5, 17–19], epilepsy [20], cancer [21], kidney diseases [22], infectious diseases [23], metabolic syndrome [24] or cardiovascular diseases [25], simultaneous modulation of different molecular targets that form a network of interconnections by a single molecule is more advantageous than the use of multiple highly specific ligands in polytherapy [9, 10, 26, 27].

## Classical polypharmacotherapy versus modern polypharmacology

In light of the briefly outlined assumptions of polypharmacology, it should be considered whether the implementation of its theoretical premises will overcome the limitations of traditional polypharmacotherapy and ultimately lead to improvements in the effectiveness of treatment of many diseases. The most important differences between “modern” polypharmacology and “classical” polypharmacotherapy are collected in Table 1.

**Table 1:** Most significant differences between polypharmacotherapy and polypharmacology (based on several studies [5–10, 12, 13, 17, 25, 26])

Feature	Polypharmacotherapy	Polypharmacology
Definition	based on multiple mono-target active pharmaceutical ingredients either used in common dosage forms or in fixed-dose combinations	based on a single active pharmaceutical ingredient that modulates multiple molecular targets simultaneously
The number of useful combinations	quite limited by the risk of drug-drug interactions, possible side effects or by technological difficulties in obtaining adequately stabile pharmaceuticals (e.g., single-pill combinations)	*theoretically* unlimited on the basis of proper selection and optimization of the structure of the molecule; *in practice*, obtaining ligands based on two to five pharmacophores is easiest
Off-target effects	possible (of each active pharmaceutical ingredient)	possible (of the multi-target directed ligand only)
Risk of drug-drug interactions	relatively high (multiple active pharmaceutical ingredients used in combination)	relatively low (one active substance only)
Pharmacokinetic profile	often difficult to predict, also for single-pill combination therapy	more predictable (especially for rationally designed multi-target directed ligands)
Drug distribution to target tissues	simultaneous administration of multiple mono-target active pharmaceutical ingredients does not ensure the uniformity of their distribution to target tissues	administration of a multi-target directed ligand leads to a uniform distribution to target tissues
Dosing regimen	may be complicated (e.g., several tablets once or even several times daily), which negatively affects patient compliance	relatively simple (e.g., one tablet once or several times daily)
Costs of treatment	purchase of multiple drugs for the treatment of a single condition represents a significant financial burden on either the patient or the healthcare system	the costs of purchasing one drug, with multidirectional effects, can be significantly lower than the costs of traditional polytherapy
Clinical trials	approval of a new polytherapy requires tests of each drug in a separate clinical trial and then tests of the combination as well (which is expensive and time-consuming)	the costs of clinical trials of a single drug candidate might be significantly lower than the sum of the costs of testing components of a new polytherapy

Most of the guidelines for the management of chronic diseases, based on the best available scientific evidence from clinical research, indicate the adequacy and effectiveness of using traditional polytherapy. On the other hand, the occurrence of a multifactorial disease depends both on the individual genetic predisposition and on environmental determinants such as nutrition, lifestyle or pollution. The exact cause of diseases such as primary hypertension is still not fully clear, despite the many efforts made in this field. Moreover, the occurrence of multiple multifactorial polytherapy-requiring diseases in a single patient, as, for example, in the elderly, further complicates the use of traditional therapeutic regimens. Indeed, the increase in the number of drugs used simultaneously, regardless of the clinical rationale, inevitably translates into an increased risk of side effects [26, 28]. Their consequences can potentially be either minor disturbances that reduce the patient's comfort of life or even dangerous iatrogenic complications requiring hospitalization [29]. Polypharmacology would be advantageous in this situation.

The combination of multiple activities in a single molecule would reliably contribute to a reduction of the number of drugs taken simultaneously. This entails advantages not only in terms of therapy efficacy, but also by improving the predictability of the pharmacokinetic profile. Reducing the risk of drug-drug interactions undoubtedly increases its safety [13, 26, 30]. When rationally designed MTDLs become widely available and cheaper therapeutic alternatives, this will also lead to the optimization of therapy costs, both on a macro (costs for the healthcare system) and micro (costs for individual patients) level [9, 31]. Moreover, the convenience of using one formulation instead of several ones would undoubtedly be reflected by an improved treatment efficacy due to improved compliance [5, 8]. This is already evident, for example, in the case of single pill combination (SPC) formulations that have been on the market for years, used in polytherapy of primary hypertension [5, 32]. A combination of the active pharmaceutical ingredients in one molecule might be even superior to traditional polytherapy based on SPC formulations. The novel 3D printing technologies might be also found advantageous in order to deliver individualized, patient-convenient oral drug dosage forms, especially in pediatric and geriatric populations [33–35].

## Polypharmacology–evolution or revolution to well-established practices?

Back at the beginning of the twenty-first century, the search for new lead structures was based on the aforementioned assumption: one drug → one target → one disease [12, 36]. However, it turned out that it could not fully address extremely important clinical challenges. Firstly, as a result of the undeniable advances in knowledge that have been made in medical science in recent decades, new possible targets have emerged. Secondly, as a result of increasingly widespread access to drugs, resistance to previously used pharmaceutics has developed. Therefore, there is a need to create effective therapies for multifactorial diseases and to find ways to overcome resistance to pharmacotherapy (particularly relevant in infectious diseases, neurodegenerative and psychiatric disorders, metabolic/cardiovascular diseases, and cancer).

**Fig. 2:**
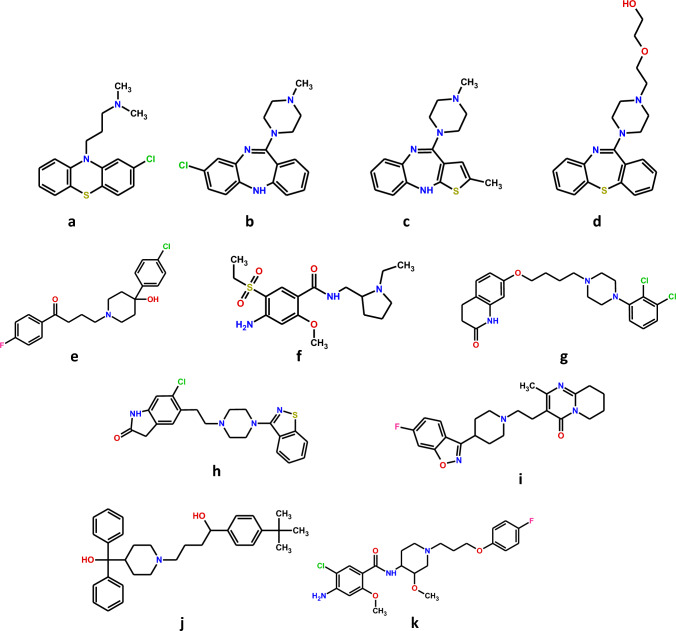
Chemical structures of small molecule drugs discussed in the text. **a** chlorpromazine;** b** clozapine; **c** olanzapine; **d** quetiapine; **e** haloperidol; **f** amisulpride; **g** aripiprazole; **h** ziprasidone; **i** risperidone; **j** terfenadine; **k** cisapride

Clinical observations and post-marketing drug safety monitoring have been often the driving force behind the development of new approaches to the design and synthesis of new drug candidates. For example, in patients taking antipsychotics, significant side effects (including extrapyramidal movement disorders) of phenothiazines like chlorpromazine (Fig. 2a) and butyrophenones like haloperidol (dopamine D_2_ receptor antagonists; Fig. 2e) have been observed. This contributed to the development of new drugs (i) with preferential action on limbic and/or presynaptic D_2_ receptors (amisulpride; Fig. 2f) [37], (ii) with partial agonistic activity at D_2_ receptors (aripiprazole; Fig. 2g) [38] or (iii) of drugs that, in addition to D_2_ receptors, target other receptors. Examples are ziprasidone (Fig. 2h, also described in detail below) and risperidone (Fig. 2i) that block 5-HT_2_ receptors with higher affinity than D_2_ receptors, thereby counteracting the extrapyramidal side effects to some extent. A higher affinity for 5-HT_2_ than for D_2_ receptors and a relatively low or even missing liability to extrapyramidal symptoms is also typical for clozapine (Fig. 2b), olanzapine (Fig. 2c) and quetiapine (Fig. 2d) [39], which, unlike ziprasidone and risperidone, have a chemical structure resembling that of the phenothiazines. Amisulpride, aripiprazole, and the latter drugs belong to the group of the second-generation (atypical) antipsychotics, which have been successfully implemented into psychiatric practice [7, 38]. The example of atypical antipsychotics distinctly shows how blurred the boundaries between classical and polypharmacological approaches to the synthesis of new drug candidates are. In both cases, it is extremely important to rationalize the activity of the active compound already at the stage of designing its structure and planning its spectrum of activity to derive the greatest possible therapeutic benefit while avoiding significant side effects [7].

Not all undesirable activities of drugs have so far been predictable at the stage of their design and synthesis. Until the 1990s, this was impossible as a result of the lack of accurate and efficient testing methods [12]. Despite the continuous progress in the field of genomics and proteomics and the development of computational methods [40], it is still challenging nowadays to determine all the potential targets to which a molecule can possibly bind [8]. The molecular basis for the adverse effects of drugs with already well-established therapeutic position is being discovered in most cases spontaneously, often many years after the registration procedure has been completed. In recent years, for example, it has been proven that molecules with basic nitrogen centers surrounded by aromatic or hydrophobic groups, such as terfenadine (Fig. 2j) or cisapride (Fig. 2k), show antagonism at potassium voltage-gated ion channel, encoded by the *KCNH2* gene, also known as human ether-à-go-go-related gene (*hERG*). Blockade of this channel leads to life-threatening ventricular tachyarrhythmias [8]. The hyperesthesia, i.e., increased sensitivity to touch, pain, pressure, and thermal sensations, occurring after treatment with the anti-migraine drug zolmitriptan was explained by its agonist activity on the serotonin 5-HT_7_ receptor. The amino acid sequence of the latter receptor is 26% identical to the sequence of 5-HT_1B_ and 5-HT_1D_ receptors, the primary targets of this drug [41].

The idea of MTDLs is still relatively new. However, this does not mean that substances with such properties have not been implemented into clinical and ambulatory use much earlier. Nevertheless, this was more the result of the "luck" of the researchers, rather than the consequence of careful planning and intensive optimization of the structure of the molecule [7]. Retrospective analyses conducted in recent years have shown the existence of beneficial pleiotropic effects, dependent on the simultaneous modulation of multiple targets, for statins used in the treatment of hypercholesterolemia and metformin, a first-line drug for the treatment of type II diabetes mellitus [7].

## De novo synthesis of multi-target-directed ligands

**Fig. 3:**
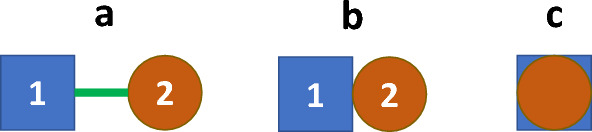
Schematic diagram of the three basic concepts for de novo synthesis of multi-target-directed ligands: **a** linked pharmacophores; **b** fused pharmacophores; **c** merged pharmacophores [7, 9, 12, 18, 19, 22]

Multi-target-directed ligands combine two or more pharmacophores within their structure, that is, fragments of a molecule with a specific spatial arrangement that have the ability to bind to a receptor (or another target) and exhibit biological activity. Figure 3 shows three different ways to create new MTDLs.

The easiest way to obtain such substances is the connection of two or more pharmacophores together using a linker (Fig. 3a). Depending on the needs, the linker used might be more or less hydrophilic. It might be both stable in vivo, as well as degradable through hydrolysis (spontaneous, caused by the change of pH of the environment, or hydrolysis catalyzed by a specific enzyme). Thus, the choice of the linker strongly affects the pharmacokinetic properties of such conjugates and determines, how the MTDL interacts with its molecular targets [9]. The undoubted advantages of linked pharmacophores are that they can be obtained relatively easily and that there is no need for excessive optimization of molecular geometry. Nevertheless, high molecular weight might reduce bioavailability or limit the ability to penetrate through the cell membrane [7, 22].

**Fig. 4:**
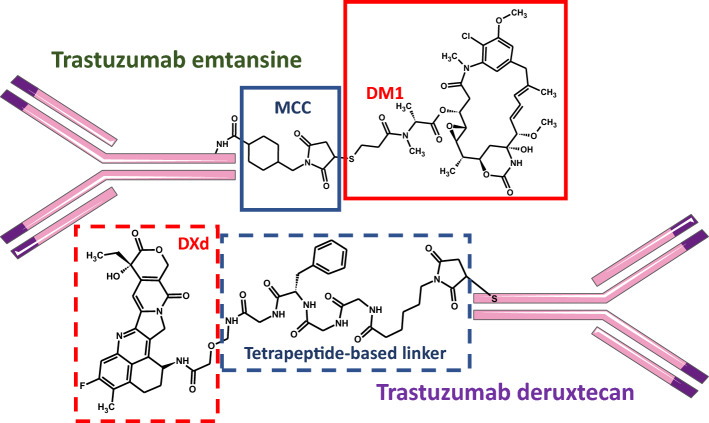
Trastuzumab emtansine and trastuzumab deruxtecan–examples of multi-target-directed ligands (MTDLs) with *linked* pharmacophores. *Upper part of the figure*. Trastuzumab (a humanized IgG1 monoclonal anti-HER2/neu antibody) is covalently bound to the microtubule inhibitor DM1 (emtansine, a derivative of maytansine; red rectangle) via the stable thioether linker MCC (4-[N-maleimidomethyl]cyclohexane-1-carboxylate; blue rectangle); an average of 3.5 DM1 molecules is conjugated to each trastuzumab molecule (based on: Kadcyla® Summary of Product Characteristics [42]). *Lower part of the figure*. Here trastuzumab is covalently bound to the topoisomerase inhibitor deruxtecan (DXd, red box, broken line) via a cleavable tetrapeptide-based linker (blue box, broken line); an average of 8 DXd molecules is conjugated to each trastuzumab molecule [57]

Examples of linked pharmacophores include antibody–drug conjugates (ADC). *Trastuzumab emtansine* is an anticancer drug used to treat HER2-positive metastatic breast cancer (Fig. 4). It consists of trastuzumab, a humanized anti-HER2/neu immunoglobulin, and the cytostatic drug emtansine. HER2 is over-expressed in ~ 20% of patients with breast cancer. This subtype is associated with a poor outcome but, on the other hand, HER2 can be targeted by trastuzumab. After the trastuzumab domain binds to its target, the dimerization of HER2 receptors will be inhibited and this process will eventually lead to suppression of tumor growth. Simultaneously emtansine penetrates into the tumor cells and induces their apoptosis [42]. The antibody also determines the selectivity of the cytostatic agent, which reaches only breast cancer cells, on the surface of which the HER2/neu receptor is overexpressed. Limitation of the systemic effects of cytotoxic substances as much as possible and focusing on their activity only on those cells that have undergone malignant transformation is the main premise and at the same time the greatest advantage of targeted anticancer therapy [8].

An alternative to the above-described combination of pharmacophores is their fusion, which means a direct combination by covalent bonding with the omission of linker groups (Fig. 3b). Fused pharmacophores allow obtaining compounds of smaller molecular weight than linked ones. Unfortunately, there may be still a high lipophilicity of such hybrid molecules with disadvantages in biopharmaceutical (relatively low bioavailability after oral administration) and technological aspects (difficulties in obtaining aqueous solutions for e.g., parenteral pharmaceutic preparations) [7, 9, 22].

**Fig. 5:**
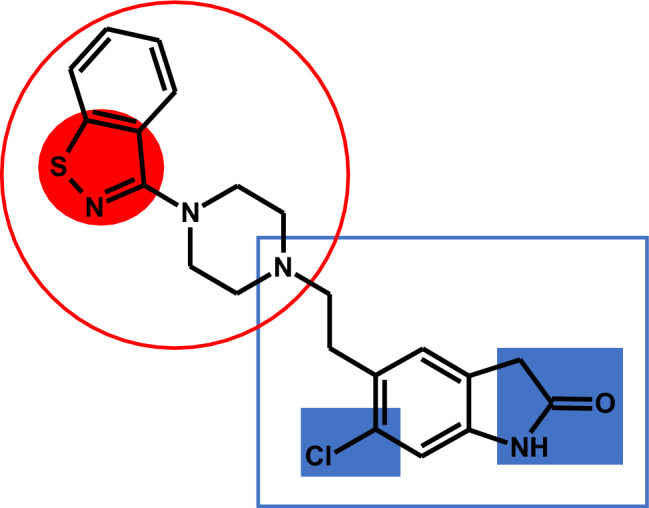
Ziprasidone—example of a multi-target-directed ligand (MTDL) with *merged* pharmacophores. The figure shows the chemical structure of ziprasidone with the merged dopamine (blue rectangle) and 5-HT_2_ (red circle) pharmacophores. Note that the chemical structure has been modified/optimized. In the dopamine part, the benzene ring is substituted with chlorine and a five-membered β-lactam ring replaces the two hydroxy functionalities of dopamine (shadowed). In the 5-HT_2_ part, the benzene ring next to piperazine is replaced by a isothiazole ring (shadowed). The figure was based on scheme 6C in the paper by Proschak et al. 2019 [7]

The highest degree of sophistication is represented by merged pharmacophores, i.e., MTDLs in which the pharmacophores share a common fragment of molecular structure. The principle is shown in Fig. 3c.  Fig. 5 shows ziprasidone as an example. Merged MTDLs are often compared to a kind of "amalgam" in which each domain responsible for the individual activity is difficult to discern at first glance. Merged pharmacophores are likely to achieve the desired low molecular weight, but they also require advanced optimization of the molecular geometry. In addition, at the stage of their design, it is necessary to exclude (very likely due to the promiscuous nature of these structures) their affinity to anti-targets [7, 9, 22].

The process of selecting proper biological targets for the three main types of MTDLs can also pose considerable problems. It requires both the determination of the role they play in the pathogenesis of a given condition, as well as the careful analysis of the possible effects of their inhibition or stimulation. It is suggested that proteins exhibiting molecular targets should have the adequate ability for modulation by a small-molecule ligand. Preference is given to proteins within families the representatives of which are targets of already used drugs (e.g., kinase inhibitors used in cancer treatment) [9].

## Examples of MTDLs introduced before 2022

In Table 2 we collected examples of Food and Drug Administration (FDA)- or European Medicines Agency (EMA)-approved MTDLs directed against infectious diseases and cancer. We did not include all the potential indications, but only those mentioned in the respective Summary of Product Characteristics.

**Table 2:** Examples of anti-infective and antineoplastic multi-target drugs introduced before 2022

International nonproprietary name (brand name)	Short description	Molecular mechanisms	Indications	References
Ozenoxacin (Ozanex®, Xepi®)	quinolone antibiotic	(–) DNA gyrase (topoisomerase II)	impetigo caused by *Staphylococcus aureus* or *Streptococcus pyogenes*	[23, 43]
		(–) topoisomerase IV		
Delafloxacin (BAXDELA®, Quofenix®)	fluoroquinolone antibiotic	(–) DNA gyrase (topoisomerase II)	acute bacterial skin and skin structure infections; community acquired pneumonia in adults	[23, 44]
		(–) topoisomerase IV		
Cefiderocol (Fetroja®)	cephalosporin antibiotic	(–) PBP	infections caused by Gram-negative aerobic bacteria in adults	[23, 45]
		Fe^3+^ (chelating)		
Axitinib (Inlyta®)	multi-kinase inhibitor	(–) VEGFR-1	advanced renal cell carcinoma in adult patients after failure of prior treatment with sunitinib or a cytokine	[48]
		(–) VEGFR-2		
		(–) VEGRF-3		
Cabozantinib (Cabometyx®)	multi-kinase inhibitor	(–) MET	renal cell carcinoma; hepatocellular carcinoma; differentiated thyroid carcinoma	[9, 49]
		(–) VEGFR-2		
		(–) RET		
		(–) ROS1		
		(–) TYRO3		
		(–) MER		
		(–) KIT		
		(–) NTRK2		
		(–) FLT3		
		(–) TIE-2		
Sunitinib (Sutent®)	multi-kinase inhibitor	(–) VEGFR-1 (–) VEGFR-2 (–) VEGFR-3	gastrointestinal stromal tumor; metastatic renal cell carcinoma; pancreatic neuroendocrine tumors	[50]
		(–) PDGFRα (–) PDGFRβ		
		(–) KIT		
		(–) RET		
		(–) FLT-3		
		(–) CSF1R		
Brentuximab vedotin (Adcetris®)	antibody–drug conjugate	antibody (b) CD30	Hodgkin lymphoma; systemic anaplastic large cell lymphoma; cutaneous T-cell lymphoma	[51]
		vedotin (–) polymerization of microtubules		
Sacituzumab govitecan (Trodelvy®)	antibody–drug conjugate	antibody (b) Trop-2	unresectable or metastatic triple-negative breast cancer	[52]
		govitecan (–) topoisomerase I		

*CD30*, cluster of differentiation 30; *CSF1R,* colony stimulating factor 1 receptor; *FLT3,* fms related receptor tyrosine kinase 3; *KIT,* receptor tyrosine kinase; *MER*, receptor tyrosine kinase; *MET (HGFR)*, hepatocyte growth factor receptor (receptor tyrosine kinase); *NTRK2,* neurotrophic receptor tyrosine kinase 2; *PDGFRα or -β*, platelet-derived growth factor receptor α or β (receptor tyrosine kinases); *PBP*, penicillin-binding proteins; *RET*, receptor tyrosine kinase; *ROS1,* receptor tyrosine kinase; *TIE-2,* angiopoietin receptor; *Trop-2,* tumor-associated calcium signal transducer 2; *TYRO3,* protein tyrosine kinase; *VEGFR-1/-2/-3*, vascular endothelial growth factor receptor-1/-2/-3; (—), blocks; (b), binds to

**Fig. 6:**
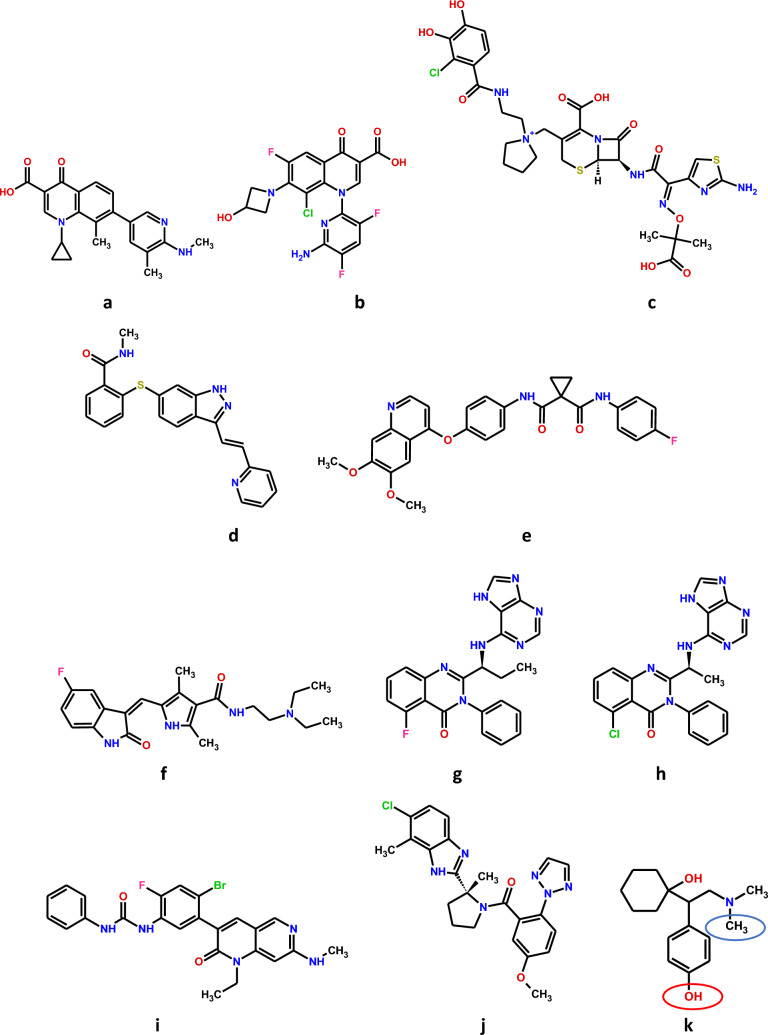
Chemical structures of recently marketed multi-target-directed ligands (MTDLs), discussed in the text: **a** ozenoxacin; **b** delafloxacin; **c** cefiderocol; **d** axitinib; **e** cabozantinib; **f** sunitinib; **g** idelalisib; **h** duvelisib; **i** ripretinib; **j** daridorexant; **k** desvenlafaxine. Desvenlafaxine is the O-demethylated (main) metabolite of venlafaxine formed by the CYP2D6 pathway (red ellipsis). N-Demethylation of venlafaxine is a minor metabolic pathway and occurs via CYP3A4, CYP2C9, and CYP2C19 (blue ellipsis) [71]

*Ozenoxacin* (Fig. 6a) is a new quinolone antibiotic, approved in 2017 by the FDA. Its special feature is the absence of a fluorine atom in the molecule. Ozenoxacin simultaneously inhibits topoisomerase IV and DNA gyrase, thereby disrupting bacterial DNA replication and transcription. It is not a substrate for efflux pumps that actively remove xenobiotics from microbial cells. As a result, it shows an increased efficacy against fluoroquinolone-resistant bacteria. Ozenoxacin is currently available on the US and Canadian market as a 1% cream for topical use [23, 43]. The same mechanism of action is shared by *delafloxacin* (Fig. 6b) [23, 44]. *Cefiderocol* (Fig. 6c), on the other hand, is a representative of modern modified β-lactams. In addition to the characteristic cephem nucleus, it has a siderophore side chain in its structure. Thanks to this, it is able to chelate extracellular Fe^3+^ ions. The complexes formed in this way are translocated into the intermembrane space of Gram-negative bacteria cells via their own siderophore uptake mechanisms. Like the other representatives of this group of antibiotics, cefiderocol can also penetrate the bacterial cell by diffusion through the porin channels of the outer cellular membrane. Cefiderocol, like other β-lactams, binds to penicillin-binding proteins (PBPs), mainly transpeptidase, thereby inhibiting bacterial cell wall synthesis, which results in cell lysis [23, 45].

The effect of trastuzumab emtansine against HER2/neu has already been discussed. *HER2* is an example of a proto-oncogene. Such proto-oncogenes are typically associated with tyrosine kinase activity and receptor protein tyrosine kinases represent an extremely typical target for small molecule antitumor drugs. At the beginning of 2023, no less than 40 compounds of that class are available [46] and some of them are multikinase inhibitors (Table 2), leading to lower effective dose, fewer side effects, and lower risk of drug resistance in comparison to single kinase inhibitors. Such a synergistic effect is an undeniable therapeutic benefit. The use of these multi-point interacting drugs might therefore contribute to overcoming resistance to traditional antineoplastic pharmacotherapy and improve the prognosis of the most severely ill patients. It is also worth noting that, in contrast to single-kinase inhibitors, multi-kinase inhibitors are considered MTDLs by many authors, and are found in line with the concept of polypharmacology, despite many of them act on targets that share high structural homology [9, 47]. *Axitinib* (Fig. 6d) potently blocks three isoforms of the vascular endothelial growth factor receptor (VEGFR-1, VEGFR-2, VEGFR-3). By doing so, it inhibits the process of pathological angiogenesis, and therefore also tumor growth and metastasis [48]. *Cabozantinib* (Fig. 6e), on the other hand, in addition to inhibiting angiogenesis, blocks other tyrosine kinases the activation of which is associated with tumor growth, pathological bone remodeling, drug resistance, and metastasis [9, 49]. *Sunitinib* (Fig. 6f), like axitinib, is a potent inhibitor of neoangiogenesis. In addition, due to its inhibitory effects on numerous other tyrosine kinases, it reduces the ability of cancer cells to grow and metastasize [50].

*Brentuximab vedotin* is an antibody–drug conjugate, another representative of the linked pharmacophores discussed earlier. It combines a monoclonal antibody (recombinant chimeric IgG1 immunoglobulin) that recognizes the Hodgkin lymphoma cell surface marker CD30 with monomethyl auristatin E (MMAE), which manifests cytotoxic effects (inhibits tubulin polymerization and formation of microtubules). After binding of the ADC to CD30, the ADC-CD30 complex is internalized and then MMAE is released via proteolytic cleavage (Table 2) [51]. *Sacituzumab govitecan* contains a humanized monoclonal anti-Trop-2 antibody. Trop-2 is a glycoprotein with an increased expression on the surface of a variety of cancer cells. Sacituzumab govitecan, after binding to Trop-2, is internalized. Then the SN-38 molecule (an active metabolite of irinotecan), which is covalently attached to the antibody via a cleavable linker, inhibits topoisomerase I. This results in impaired DNA replication and ultimately leads to cell apoptosis (Table 2) [52].

## New multi-target drugs in 2022

Some examples of successfully marketed MTDLs have been described above and in the reviews quoted [6–10]. In order to get an idea of whether MTDLs really play a significant role in contemporary pharmacology we considered the new drugs marketed in Germany in 2022 [53]. Those drugs had previously been approved by the EMA, which represents the counterpart of the FDA responsible for the USA. EMA is in charge of the 27 countries of the European Union. Among the 49 new drugs, 10 can be classified as drugs with multi-targeting properties (Table 3). Seven drugs are indicated for the treatment of cancer and other malignant neoplasms (#1-#7), another two for the treatment of disorders of the central nervous system (#8 and #9), and the final one (# 10) for the treatment of eye disease.

**Table 3:** Ten new multi-target drugs introduced in Germany in 2022

International nonproprietary name (brand name)	Short description	Molecular mechanisms	Indication(s)	References
Duvelisib (Copiktra®)	phosphatidylinositol-3-kinase inhibitor	(–) phosphatidylinositol-3-kinase–δ isoform	third-line therapy of adults with relapsed or refractory chronic lymphatic leukemia or follicular lymphoma	[54]
		(–) phosphatidylinositol-3-kinase–γ isoform		
Ripretinib (Qinlock®)	tyrosine kinase inhibitor	(–) KIT kinases (broad range)	fourth-line therapy of locally advanced or metastatic gastrointestinal stromal tumor	[55]
		(–) PDGFRα kinase		
Amivantamab (Rybrevant®)	bispecific antibody	(–) EGFR	advanced non-small cell lung cancer in adult patients with exon20 insertion of EGFR who had received a platinum-containing chemotherapy	[56]
		(–) MET		
Trastuzumab deruxtecan (Enhertu®)	antibody–drug conjugate	antibody (b) HER2/neu	unresectable or metastatic HER2-positive breast cancer in adult patients who have received one or more prior anti-HER2-based regimens	[57]
		deruxtecan (–) topoisomerase I		
Enfortumab vedotin (Padcev®)	antibody–drug conjugate	antibody (b) nectin-4	locally advanced or metastatic urothelial cancer in adult patients who had previously received a platinum-containing chemotherapy and a programmed death receptor-1 or programmed death-ligand 1 inhibitor	[58]
		monomethyl auristatin E (–) polymerization of tubulin		
Mosunetuzumab (Lunsumio®)	bispecific antibody	(b) CD20 on malignant B cells	third-line treatment of relapsed or refractory follicular lymphoma in adults who had received at least two prior systemic therapies	[59]
		(b) CD3 on cytotoxic T cells		
Tebentafusp (Kimmtrak®)	bispecific fusion protein	monoclonal T cell protein with antibody fragment (b) gp100 of melanoma cells	HLA-A*02:01-positive adult patients with unresectable or metastatic uveal melanoma	[60]
		antibody against CD3 (+) polyclonal T cells		
Daridorexant (Quviviq®)	antagonist of two G protein-coupled receptors	(–) OX_1_ receptor	adults sufferring from insomnia that has been lasting for ≥ 3 months and has a marked impact on daytime activity	[62]
		(–) OX_2_ receptor		
Desvenlafaxin (Desveneurax®)	inhibitor of two amine transporters	(–) serotonin reuptake	major depression in adults	[63]
		(–) noradrenaline reuptake		
Faricimab (Vabysmo®)	bispecific antibody	(–) vascular endothelial growth factor A	neovascular (wet) age-related macular degeneration or diabetic macular edema	[64]
		(–) angiopoietin-2		

*CD3, CD20,* cluster of differentiation 3, 20; *EGFR*, epidermal growth factor receptor (receptor tyrosine kinase); *gp100,* glycoprotein 100, occurring in melanocytes; *HER2/neu*, receptor tyrosine kinase within the erbB-2 family; *HLA-A,* human leukocyte antigen A; *KIT*, receptor tyrosine kinase; *MET (HGFR),* hepatocyte growth factor receptor (receptor tyrosine kinase); *PDGFRα,* platelet-derived growth factor receptor α (receptor tyrosine kinase); (+), activates; (–), blocks; (b), binds to

The drugs indicated for the treatment of malignant neoplasms may be arbitrarily sub-classified into three groups. Drugs #1, #2, and #3 target proto-oncogene protein or kinase activity. In the case of #3, two proto-oncogenes are inhibited by a bispecific antibody, and, in the case of #1 and #2, kinase activity is inhibited by organic compounds with a low molecular weight (“small molecules”). In the second and third groups, one part of the drug molecule identifies malignant cells whereas the other one severely disables tumor cell function/integrity either by a cytostatic (#4 and #5) or immunologic mechanism (#6 and #7), respectively.

Chronic lymphocytic leukemia and the very rare follicular lymphoma are B cell lymphomas, treatment of which is necessary only if they show symptoms. Both cytostatic drugs and drugs specifically targeting B cells are used for therapy. One of the drugs of the latter category is idelalisib (Fig. 6g), which inhibits the δ-isoform of the phosphatidylinositol-3-kinase, which plays an important role in the development and function of B cells. In addition, *duvelisib* (#1; Fig. 6h) also inhibits the γ-isoform of this enzyme, which is involved in the differentiation and migration of crucial tumor support cells in the tumor microenvironment (Table 3). Some studies suggest that duvelisib provides improved efficacy over idelalisib [54].

In the gastrointestinal stromal tumor with an incidence of ~ 1 case per 100,000 per year the proto-oncogenes *KIT* and *PDGFRα* are important. For the treatment of advanced and metastatic stages, the tyrosine kinase inhibitor imatinib is indicated [55]. However, this drug, the first tyrosine kinase inhibitor at all [46], is not active in all patients since the tumor shows a primary resistance or undergoes secondary resistance due to mutations of the *KIT* and *PDGFRα* genes. Similar problems may occur if sunitinib and regorafenib are used as second- and third-line therapy, respectively; the latter drugs may also lead to severe side effects. *Ripretinib* (#2; Fig. 6i) is a safe and effective new kinase inhibitor, targeting *KIT* and *PDGFRα* mutations and resistance (Table 3) [55].

Epidermal growth factor receptor (EGFR) and hepatocyte growth factor receptor (MET or HGFR) are another two receptor tyrosine kinases. They play a role in non-small cell lung cancer (NSCLC), but not in small cell lung cancer (SCLC) [56]. For the treatment of this cancer entity, tyrosine kinase inhibitors are useful, but frequently lose efficacy due to secondary mutations in the *EGFR* gene, e.g., the exon20 insertion. In this case, the bispecific monoclonal antibody *amivantamab* (#3), which is directed against EGFR with exon20 insertion and against MET, can be used (Table 3) [56].

The ADC trastuzumab emtansine, which is indicated for the treatment of HER2-positive breast cancer, has already been discussed (Fig. 4). In *trastuzumab deruxtecan* (#4) the topoisomerase I inhibitor deruxtecan serves as the cytostatic drug (Fig. 4). Each antibody is linked to 8 molecules of deruxtecan as opposed to only ~ 3.5 in the case of emtansine. Once the trastuzumab domain binds to the HER2/neu receptor, the entire antibody–drug complex undergoes internalization. The mechanism of action of deruxtecan is similar to that of the above-mentioned emtansine, but its potency is almost 10 times higher [57]. Moreover, binding between antibody and deruxtecan (as opposed to emtansine) is cleavable and the released deruxtecan can diffuse outside of the targeted cell and destroy also tumor cells, *not* expressing HER2, in the vicinity. Since trastuzumab can lead to interstitial lung disease special precautions must be met in order to avoid this potentially lethal side effect (Table 3) [57].

*Enfortumab vedotin* (#5), which is indicated for the treatment of advanced or metastatic urothelial cancer, is another example of an ADC; the cytotoxic compound monomethyl auristatin E (MMAE) is linked to a human anti-nectin-4 antibody via a protease-cleavable linker. Nectin-4 is an adhesion protein present on the surface of urothelial cancer cells. After the binding of the conjugate to nectin-4 expressing cells, the complex formed by the antibody–drug conjugate and nectin-4 is internalized and then MMAE is released, which leads to disruption of the microtubular network and eventually apoptotic cell death [58].

Follicular lymphoma, a malignant neoplasm of B cells, which is prone to frequent relapses, has already been mentioned under duvelisib. Another new drug indicated for the third-line treatment of advanced stages of this neoplasm is *mosunetuzumab* (#6). This bispecific antibody identifies malignant B cells by binding to their CD20 domain and activates cytotoxic T cells by binding to their CD3 domain. It is worth noting that mosunetuzumab is considered a *conditional* agonist, which means that only its simultaneous binding to CD20 on B-cells and CD3 on T-cells (creating an *immunologic synapse*, which engages both arms of mosunetuzumab) will ultimately lead to the activation of cytotoxic T cells. As a result, perforin and granzymes, released from activated T-cells, will eventually lead to B cell lysis and cell death (Table 3) [59].

*Tebentafusp* (#7), a bispecific fusion protein, is another example of an MTDL which (i) identifies malignant cells and (ii) activates the immune system against those malignant cells. The drug is the first treatment option for advanced uveal melanoma, a rare and aggressive type of melanoma in patients with a special HLA-A antigen (Table 3). In detail, one part of the drug targets a specific HLA-A*02:01/gp100 complex on uveal cells, whereas the other binds to the CD3 domain of CD4 + /CD8 + effector and memory T cells that, in turn, induces tumor cell death [60].

Another two drugs act against disorders of the central nervous system. Insomnia is a very frequent disorder and many hypnotics are available for its treatment but have disadvantages including limited efficacy (e.g., plant extracts), acute toxicity (e.g., antihistamines) and abuse liability (e.g., benzodiazepines). So, there is an urgent need for new therapeutic strategies. *Daridorexant* (#8; Table 3; Fig. 6j), a dual orexin receptor antagonist, blocks the two G protein-coupled receptors (OX_1_ and OX_2_) activated by orexin A and B. However, although OX_1_ and OX_2_ share a 64% common amino acid sequence, antagonists of these receptors were classified into two separate groups: single (selective) antagonists and dual antagonists. The latter may correspond to the polypharmacology concept [61]. Daridorexant represents an entirely new therapeutic principle. The orexins are peptides formed in the hypothalamus and promote wakefulness. In clinical studies, daridorexant reduced both the latency to persistent sleep (parameter for sleep onset) and the wake time after sleep onset (parameter for sleep maintenance). The secondary endpoint, i.e., self-reported subjective total sleep time, was also favourably influenced by daridorexant [62]. It is, of course, too early to decide whether daridorexant can replace traditional hypnotics.

For the treatment of depression, many classes of drugs are available. The tricyclic antidepressants, which became available in the fifties of the last century, are “dirty” drugs, since they do not only block the neuronal serotonin and noradrenaline transporter, but also a series of G protein-coupled receptors, including muscarinic, histamine H_1_ and α_1_-adrenergic receptors. The latter three effects are not necessary for an antidepressant effect, and selective serotonin (SSRI) and selective serotonin plus noradrenaline re-uptake inhibitors (SSNRI or SNRI) retain the antidepressant effect. One of the SNRIs is venlafaxine and its active metabolite *desvenlafaxin *(#9; Fig. 6k) has been marketed as its own drug entity in 2022 (Table 3) [63].

The last drug is *faricimab* (#10), which is indicated for the treatment of neovascular (wet) age-related macular degeneration and diabetic macular edema (Table 3) [64]. Age-related macular degeneration accounts for 9% of blindness worldwide and for ~ 50% of blindness and high-grade visual impairment in Germany [65]. For the treatment of the neovascular (wet) form of the disease, several intravitreally injected “antagonists” of the vascular endothelial growth factor (VEGF) A are available, including the monoclonal antibody ranibizumab, and the fusion protein aflibercept. These compounds inhibit the formation and growth of pathological blood vessels and the extravasation of blood plasma components into the retinal parenchyma [65]. The bispecific antibody faricimab is directed not only against VEGF-A but, in addition, also against angiopoietin-2. Antibodies against the latter are thought to promote vascular stability and to desensitize blood vessels to the effects of VEGF-A [64].

Whether newly introduced drugs provide a benefit is being assessed in Germany by the Joint Federal Committee (“Gemeinsamer Bundesausschuss”) consisting of representatives of physicians, dentists, hospitals, and health insurance providers. They check after six months whether a newly introduced drug has an advantage over the standard therapy. This procedure is stricter than that of the EMA, who postulates the therapeutic effect and safety of the new drug only. The additional benefit, if any, can be categorized as major, considerable, or minor [66]. The assessment procedure was initiated in 2011 and has been used 717 times until 20 March 2023. Only eleven drugs received the highest degree of a *major* additional benefit [67] and one of them is #2 (ripretinib, Table 3) [68]; administration of this drug along with best supportive care (BSC) is by far superior to placebo plus BSC used in the comparator group. It is not so rare that an additional benefit cannot be documented, e.g., if there is no significant difference between the two groups or an appropriate comparator group was not recruited by the manufacturer of the new drug; the former was true for #3 (amivantamab, Table 3) [69]. When an additional benefit cannot be demonstrated the annual treatment costs of the new drug must not exceed those of the appropriate comparator [66]. In the case of amivantanab, the manufacturer therefore decided to withdraw Rybrevant® from the German market already eight months after its introduction [70].

## Conclusion

Physicians are frequently confronted with multifactorial diseases or with the problem that resistance, e.g., of microbes or tumor cells, is developing. To overcome such challenges single selective drugs may be combined, but they may differ in their pharmacokinetic properties, interact with each other or exhibit multiple off-target effects. If the single components are administered separately (and not in a polypill), lack of compliance may become an additional problem. As a new concept, polypharmacology (*the design or use of pharmaceutical agents that act on multiple targets or disease pathways*) has gained increasing acceptance. For this purpose, pharmacophores are linked (e.g., an antibody is connected with a small organic molecule by a linker), fused (molecules connected by covalent bonding), or merged (overlap of pharmacophores). In the meantime, many multi-target drugs have been introduced. To test their contribution to the contemporary drug armamentarium, 49 drugs introduced in Germany in 2022 were checked and 10 MTDLs were identified amongst them. They comprise seven antitumor agents, including ripretinib, a multikinase inhibitor, trastuzumab deruxtecan, a conjugate of an anti-HER2/neu antibody with a cytostatic agent, and tebentafusp, a fusion protein directed against gp100 of melanoma cells plus CD3 of T cells; drugs are indicated for advanced gastrointestinal stromal tumor, breast cancer, and uveal melanoma, respectively. Another two drugs act against disorders of the brain, including daridorexant, a drug with an entirely new hypnotic mechanism, i.e. antagonism towards the orexin OX_1_ and OX_2_ receptors. Finally, faricimab, an antibody against vascular endothelial growth factor A and, in addition, angiopoietin-2, is a new option for the treatment of age-related macular degeneration.

## Data Availability

Data sharing is not applicable to this article as no datasets were generated or analyzed during the current study.

## Abbreviations

ADC: Antibody–drug conjugate
BSC: Best supportive care
CD20: Cluster of differentiation 20
CD3: Cluster of differentiation 3
CD30: Cluster of differentiation 30
CD4: Cluster of differentiation 4
CD8: Cluster of differentiation 8
COVID-19: Coronavirus disease 2019
CSF1R: Colony-stimulating factor 1 receptor
EBM: Evidence-based medicine
ECMO: Extracorporeal membrane oxygenation
EGFR: Epidermal growth factor receptor (receptor tyrosine kinase)
EMA: European Medicines Agency
FDA: Food and Drug Administration
FLT3: Fms related receptor tyrosine kinase 3
gp100: Glycoprotein 100 (occurring in melanocytes)
HER2/neu: Receptor tyrosine kinase within the erb-b2 family
hERG: Human ether-à-go-go-related gene
HLA-A: Human leukocyte antigen A
IgG1: Immunoglobulin G1
KCNH2: Voltage-gated potassium channel subfamily H member 2
KIT: Receptor tyrosine kinase
MER: Receptor tyrosine kinase
MET (HGFR): Hepatocyte growth factor receptor (receptor tyrosine kinase)
MMAE: Monomethyl auristatin E
MTDL(s): Multi-target-directed ligand(s)
NSCLC: Non-small cell lung cancer
NTRK2: Neurotrophic receptor tyrosine kinase 2
PBP(s): Penicillin-binding protein(s)
PDGFRα: Platelet-derived growth factor receptor α (receptor tyrosine kinase)
RET: Receptor tyrosine kinase
ROS1: Receptor tyrosine kinase
SARS-CoV-2: Severe acute respiratory syndrome coronavirus type 2
SCLC: Small cell lung cancer
SNRI (SSNRI): Selective serotonin plus noradrenaline reuptake inhibitors
SPC: Single-pill combination
SSRI: Selective serotonin reuptake inhibitors
TIE-2: Angiopoietin receptor
Trop-2: Tumor-associated calcium signal transducer 2
TYRO3: Protein tyrosine kinase
VEGF A: Vascular endothelial growth factor A
VEGFR-1/-2/-3: Vascular endothelial growth factor receptor-1/-2/-3
WHO: World Health Organization
